# Elements of Social Convoy Theory in Mobile Health for Palliative Care: Scoping Review

**DOI:** 10.2196/16060

**Published:** 2020-01-06

**Authors:** Jennifer D Portz, Kira Elsbernd, Evan Plys, Kelsey Lynett Ford, Xuhong Zhang, M Odette Gore, Susan L Moore, Shuo Zhou, Sheana Bull

**Affiliations:** 1 General Internal Medicine, School of Medicine University of Colorado Aurora, CO United States; 2 Colorado School of Public Health University of Colorado Aurora, CO United States; 3 Department of Cardiology Denver Health and Hospital Authority Denver, CO United States; 4 School of Medicine University of Colorado Aurora, CO United States

**Keywords:** mHealth, palliative care, caregivers, mobile apps

## Abstract

**Background:**

Mobile health (mHealth) provides a unique modality for improving access to and awareness of palliative care among patients, families, and caregivers from diverse backgrounds. Some mHealth palliative care apps exist, both commercially available and established by academic researchers. However, the elements of family support and family caregiving tools offered by these early apps is unknown.

**Objective:**

The objective of this scoping review was to use social convoy theory to describe the inclusion and functionality of family, social relationships, and caregivers in palliative care mobile apps.

**Methods:**

Using the Preferred Reporting Items for Systematic Reviews and Meta-Analyses extension for Scoping Review guidelines, a systematic search of palliative care mHealth included (1) research-based mobile apps identified from academic searches published between January 1, 2010, and March 31, 2019 and (2) commercially available apps for app stores in April 2019. Two reviewers independently assessed abstracts, app titles, and descriptions against the inclusion and exclusion criteria. Abstracted data covered app name, research team or developer, palliative care element, target audience, and features for family support and caregiving functionality as defined by social convoy theory.

**Results:**

Overall, 10 articles describing 9 individual research-based apps and 22 commercially available apps were identified. Commercially available apps were most commonly designed for both patients and social convoys, whereas the majority of research apps were designed for patient use only.

**Conclusions:**

Results suggest there is an emerging presence of apps for patients and social convoys receiving palliative care; however, there are many needs for developers and researchers to address in the future. Although palliative care mHealth is a growing field, additional research is needed for apps that embrace a team approach to information sharing, target family- and caregiver-specific issues, promote access to palliative care, and are comprehensive of palliative needs.

## Introduction

### Background

Nearly 1.7 million people will die in the United States each year from a serious chronic illness, including heart disease, cancer, and respiratory disease [1]. These patients experience significant physical and psychological symptom burden and progressive dependence on their family and caregivers [2]. For the months or years leading to death, palliative care provides an interdisciplinary and patient-family centered approach to address the physical, psychological, emotional, and spiritual suffering for patients and families [3]. The primary goal of palliative care is to improve quality of life.

Palliative care is provided by an interdisciplinary team often made up of physicians, nurses, social workers, and chaplains. Specialty services including physical therapy, occupational therapy, and music or art therapy may also be offered. Palliative care strategies target symptom management, medication management, family support and training, advance care planning and goals of care facilitation, caregiver respite, and interventions for emotional and spiritual needs of patients and family [4]. Hospice is a specific type of palliative care provided in the terminal phase of end-of-life care. Hospice provides 24-hours a day palliative care services focused on symptom management, provision of needed medical equipment, psychospiritual and emotional aspects of dying, respite care, family coaching, bereavement, and grief services. In the United States, hospice is specifically associated with the Hospice Medicare Benefit and is provided only to terminally ill patients with a life expectancy of 6 months or less who no longer seek potentially curative treatment such as chemotherapy and dialysis.

Palliative care improves patient and family reported outcomes, including quality of life and satisfaction, reductions in emergency visits and hospitalizations at end-of-life, and increased referral and length of stay with hospice services [5]. However, estimates suggest that only 3.4% of hospital admissions are referred to palliative care. Nearly 1 million patients admitted who could benefit from palliative care do not receive this specialized service [6]. Access barriers to palliative care are commonly attributed to palliative care resource availability, lack of awareness, and provider and patient and family reluctance [7].

Mobile health (mHealth), the use of mobile devices to improve health services and health outcomes, provides modern opportunities for patients and their family to engage in palliative care but is relatively underexplored. mHealth may provide access to palliative care support for patients and families that may not otherwise receive specialty palliative care or hospice services. There are mHealth palliative care apps available commercially, and health-related researchers are currently initiating app design efforts [8,9]. However, the elements of family support and family caregiving tools offered by these early apps is unknown.

On the basis of a national survey of caregivers, an estimated 40% to 65% of family caregivers are interested in using mHealth to support and monitor the health of their loved ones [10,11]; however, mHealth systems are typically designed for individual users, rather than integrating the patient’s family, friends, and social support to maximize benefit. This contradicts the behavioral science findings indicating social support is a critical construct in improving health behaviors and health outcomes. Specifically, Social Convoy Theory [12-14] is well established in the social science literature and provides a framework for understanding the complex relationships of individuals within a group of people that give and receive social support over the life cycle. The convoy can include informal supports such as family members, friends, and neighbors and formal supports such as professional caregivers. Previous research substantiates a link between social convoys and convoy relationships with improved health outcomes, reduced mortality in older populations, and quality of life among patients with serious illness [15-17]. The structure, function, and quality of one’s social convoy is associated with quality of life, the primary health outcome for palliative care. Therefore, when designing mHealth specially for palliative care, it is important to incorporate a social convoy perspective considering that the family is a key component of care. As people with serious illness increasingly rely on the support of others to help manage their health, there is a critical need to foster approaches for effective integration of the convoy in palliative care–specific mHealth.

### Objective

Although palliative care improves health outcomes and quality of life, there are barriers to accessing specialty services. Growing acceptability of mHealth offers promise in leveraging tools with family and the caregiving team to expand access. The objective of this scoping review was to describe the integration of social convoy theory in current palliative care–specific mHealth. Findings from this work will be used to inform strategies for designing mHealth interventions that are not only addressed to individual patients but also integrate their social convoy of families, friends, and caregivers.

## Methods

### Scoping Review

As little is known about palliative care mHealth, a scoping review approach was used to comprehensively review palliative care–specific mobile apps to determine the extent and nature of social convoy features available. The Preferred Reporting Items for Systematic Reviews and Meta-Analyses extension for Scoping Review (PRISMA-ScR) checklist guided the work, informing the search, selection, and evaluation of mobile apps [18]. This review is unique as it applies the PRISMA-ScR method to review both commercially available apps (offered from app stores) and research-based apps (described in the health-related scientific literature) to systematically review all current palliative care–specific apps.

### App Search and Screening

**Figure 1:**
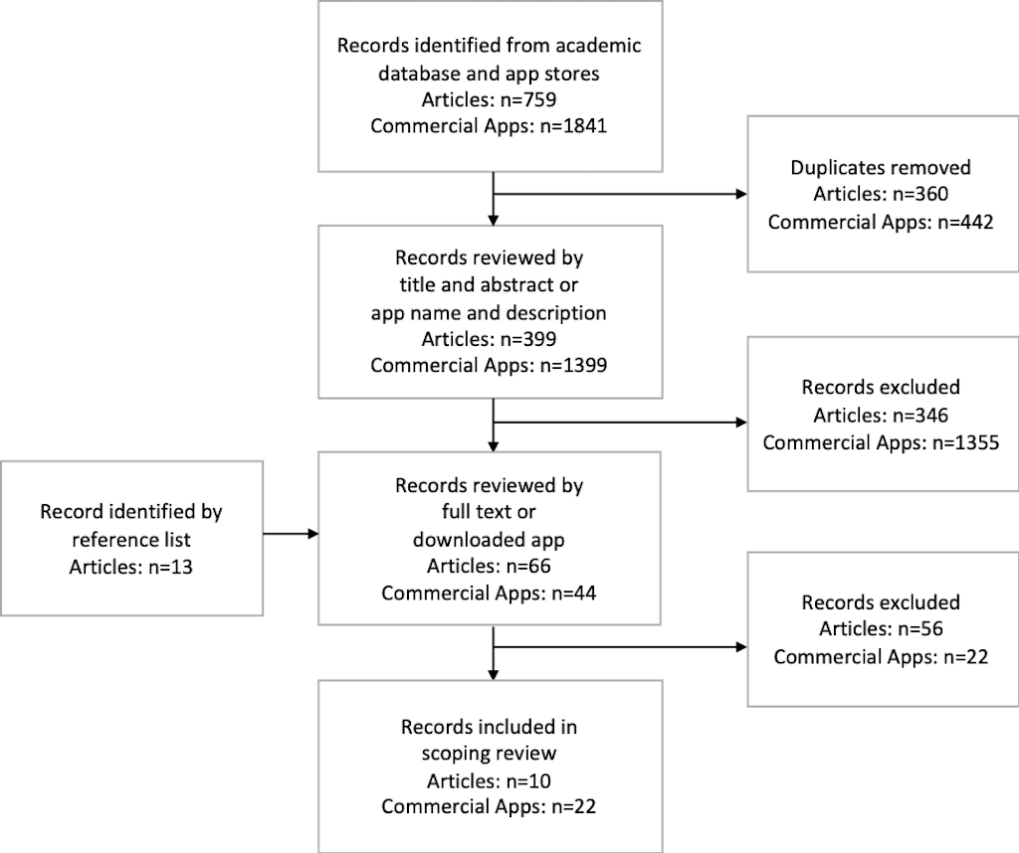
Flowchart for selection of research-based and commercially available apps.

To identify research-based apps, the search utilized 3 academic databases including PubMed, PsycINFO, and Web of Science for peer-reviewed palliative care mHealth studies. The search included empirical studies published between January 1, 2010, and March 31, 2019. The search string was restricted to search terms included in the title and abstract and included: palliative care OR hospice OR end-of-life OR terminal illness OR advance directives OR living will OR symptom OR advanced care planning OR spiritual care OR grief OR bereavement AND mobile OR smartphone OR app OR mHealth. This search string ensured that only mHealth interventions for palliative care specially were identified. Systematic review articles were not included in the final scoping review, rather the specific apps listed in the reviews’ results were assessed for inclusion. In addition to research-based apps, an electronic search in April 2019 browsed official app stores for iPhone (Apple Inc [iOS, The App Store]), Google Play (Google, LLC [Android, ChromeOS, Google Play Store]), and Amazon Appstore (Amazon Inc [Android, FireOS, Blackberry, Amazon App Store]) to identify free, commercially available palliative care apps. Identical palliative care terms were used as listed above, which have previously been used to identify mHealth interventions directed toward palliative care [8]. Duplicate articles and apps were removed before screening eligibility. A summary of search and screening process is described in Figure 1.

Mobile apps meeting the following inclusion criteria were included in the review: (1) focused on at least one element of palliative care (quality of life assessment, symptom management, family support including bereavement or grief, spiritual care, psychosocial support, decision support, and patient/family education), (2) targeted adults with serious life-limiting illness and/or their family and caregivers, and (3) offered via a mobile app, that is, articles using websites that can be accessed via a mobile device were not included in this review. The primary reasons apps were excluded from the review are as follows: not available for free, not available in English, not available in the United States, pediatric focus, provider targeted, hospice eligibility and referral only, funeral planning only, and theoretical prototypes. Articles that did not provide detailed description of the mobile app were excluded.

To determine if apps met inclusion criteria, 2 coders (JDP and KE) independently reviewed the academic articles by title and abstract and screened commercial apps by app name and description provided by the app store. The full text of articles was then downloaded and further reviewed for eligibility. Commercially available apps were downloaded, and a user account was established. The coders met regularly to review articles and commercial apps to discuss questions and possible disputes. Both coders agreed on the final apps included in the review. The search and screening process resulted in 10 articles [19-28] describing 9 individual research-based apps and 22 commercially available apps. Furthermore, none of the included research-based apps were available commercially.

### Data Extraction

Once included, information about the apps were abstracted into a Microsoft Excel spreadsheet. Abstracted data covered app name, research team or developer (individual or organization), palliative care element, target audience (patient, convoy, or both), and features for family support and caregiving functionality as defined by Social Convoy Theory. Review of research-based apps was based on the description of app features provided in the article, whereas all tabs and features of commercially available apps were reviewed for the elements listed above. Apps were considered patient-focused if features were designed primarily for patients, such as education or symptom tracking without the ability to share information captured in the app with caregivers; convoy-focused if features were designed primarily for caregivers, such as bereavement tools; and both patient and convoy-focused if app features were designed to share information and facilitate a patient-convoy relationship. Specifically, information was abstracted related to (1) convoy composition—app targets particular convoy relationships such as informal caregivers, formal caregivers, family, neighbors, friend, and/or other convoy members; (2) convoy size—the number of convoy members that can use the app; (3) personal convoy characteristics—app considerations of factors such as age, race, ethnicity, and/or gender of the patient and convoy; (4) contextual convoy characteristics—app considerations of factors such as marriage, social isolation, and/or socioeconomic status of the patient and convoy; and (5) convoy support—app facilitation between patient and convoy.

## Results

### Summary of Apps

A summary of social convoy elements in palliative care mHealth is provided in Table 1. Overall, 22 commercially available mobile apps (Table 2) and 10 articles describing 9 apps (Table 3) were selected for full review. A total of 11 of 22 apps were available through the Apple app store only, 3 through the Google store only, 7 were available through both Apple and Google stores, 1 through Google and Amazon app stores, and 1 through all 3 app stores. Apps identified in the literature were in various stages of development, which included articles describing at least a minimum viable product. A total of 6 of the 9 apps identified were prototypes undergoing acceptability and/or usability pilot testing, a further 2 apps were under evaluation in randomized controlled trials with no results yet available, and 1 article described results from a randomized controlled trial using an app-based intervention.

**Table 1:** Commercially available and research app elements (N=31).

Element		Commercial (n=22), n (%)	Research (n=9), n (%)
**Primary palliative care component**			
	Quality of life	1 (5)	0 (0)
	Psychosocial support	3 (14)	0 (0)
	Decision support	5 (23)	1 (11)
	Symptom management	15 (48)	8 (89)
	Bereavement or grief	5 (23)	0 (0)
	Patient/family education	1 (4.5)	0 (0)
**Target user**			
	Patient	8 (36)	8 (89)
	Convoy	5 (23)	1 (11)
	Both patient and convoy	9 (41)	0 (0)
**Convoy composition and size**			
	Convoy can be independent app users	4 (18)	1 (11)
	Convoy can be independent users but are not connected	3 (14)	0 (0)
	Patient shares app-generated content with convoy	10 (45)	0 (0)
	Targets patient only	5 (23)	8 (89)
Considers age, race, ethnicity, and gender		6 (27)	1 (11)
Considers marriage, social isolation, and socioeconomic status		2 (9)	0 (0)

**Table 2:** Summary of commercial apps.

Developer^a^	App name	Element of palliative care	Target users	Description
ADVault Inc	My Directives	Decision support	Patients	Patients can fill out simple medical wishes to auto-populate an advance directive that can be shared with convoy via email, text, or scanner. Allows patient to select a proxy from phone contacts and confirms understanding and willingness of proxy via email.
Ben Delaporte	ChronicPainDiary	Symptom management	Patients	Patients are offered to enter a daily pain score scaled 1-10 daily. A color-coded line graph shows scores over 1 week to 1 month to monitor changes over time.
Boston Scientific, Inc	MyPainScale	Symptom management	Both patients and convoy	Allows patient to track symptoms and share reports with convoy via text.
Center for Advancing Health	AfterShock	Patient/family education	Patient	Provides education for patients after receiving a terminal diagnosis. Ability to share information with convoy members included.
Erin Cole	LifeChest	Psychosocial support	Patient	Stores personal information about the patient (insurance, health, financial, life wishes, etc), including pictures of important documents, which can be shared with convoy members via email.
Final Thoughts LLC	Final Thoughts	Psychosocial support	Both patients and convoy	Offers exchange of legacy projects (multiple media options) at present or scheduled for the future between the patient and convoy. The patient can select who among phone contacts and app users receives legacy information.
Goodgrief Works, LLC	goodgrief	Bereavement or grief	Convoy	Grief social media app connecting people who share similar types of loss. Offers chat wall and personal instant messaging.
Infinite Monkeys LLC	Grief Support Network	Bereavement or grief	Convoy	Offers resources for grief and links to outside sources, connects to radio and YouTube, and provides social network function.
Jeremy Gonzalas	Farewell	Psychosocial support	Both patients and convoy	Provides avenue for exchange of legacy projects to specific convoy groups categorized by family, friends, work, spiritual, and financial. The patient provides passcode to convoy members to access patient-created content.
Nous Foundation	ACPDecisions	Decision support	Patient	Provide ACP tools including definitions for ACP and palliative care, steps for ACP, advance directive forms, and videos about sharing ACP preferences. The app is available in 9 languages.
Prognosis and Therapeutic Harmonization (PATH) Limited	The Fragility App	Quality of life assessment and symptom management	Convoy	Assessment of patient symptoms and function and convoy member stress and access to support completed by convoy member. Ability to send report via SMS to other convoy members or provider phone number.
SCET	Lets Think Ahead-My ACP	Decision support	Both patients and convoy	Allows the patient to enter and share end-of-life medical care decisions with family via email. Also provides assessment of conditions, experience of having a serious illness.
Scott La Counte	iGrief	Bereavement or grief	Convoy	Offers readings and quotes categorized by specific emotions.
Selesti	ButterflyProject	Bereavement or grief	Convoy	Offers resources, mindfulness activities, music, inspirational quotes, and a memory box.
Selesti	SmilesandTears	Bereavement or grief	Convoy (bereaved children)	Features include memory jar, balloon release, virtual gift giving, and diary.
Self Care Catalysts Inc	HealthStoryLines	Symptom management	Both patients and convoy	Facilitates exchange of health information, specifically symptom and mood tracking between the patient and patient-selected convoy members. Also includes appointment and medication features and syncs to sensor technologies.
SILECI Apps	Simple Symptom Tracker	Symptom management	Patients	Allows for unlimited symptoms to be tracked and notation option to describe factors that might influence the symptom. Offers graphs for reviewing symptoms over time.
Smooth Mobile, LLC	Symptom Tracker	Symptom management	Both patients and convoy	Offers symptom tracking and reports, which can be shared with convoy via Excel, Cloud, or printable PDF.
VJ Periyakoil	AdvanceDirectives Stanford University	Decision support	Patients	Stores patient medical and emergency information, organ donation, and end-of-life medical care decisions, which can be shared with convoy via email. Includes ability to add pictures or video.
Universal Projects and Tools, SL	mPalliative Care App	Symptom management	Both patients and convoy	Patients can track symptoms and share reports with convoy via text.
University of Zurich	Catch My Pain	Symptom management	Both patients and convoy	Patients can track symptoms and share reports with convoy via text.
William Palin	PaperHealth	Decision support	Both patients and convoy	Allows patients to enter and share designated health proxy and end-of-life care preferences with convoy via email or text.

^a^Commercial apps were not directly connected to health care providers or clinical electronic medical record. All apps provided at least one export function for data that could be shared with providers via the patient or caregiver.

**Table 3:** Summary of research apps.

Study	App name	Element of palliative care	Target user	Description
Agboola et al (2014) [19]; Fishbein et al (2017) [20]	CORA	Symptom management	Patients on oral anticancer medication	Provides daily educational and/or support push notifications, offers symptom reporting and strategies for symptom management, weekly symptom and activity reports, and is customizable to patient needs, abilities, and medications.
Agboola et al (2014) [21]	ePAL	Symptom management	Patients with cancer	Offers on-demand pain assessments, daily educational and/or support push notifications, multimedia resource library including psychosocial support material, prescription refills, and ability to personalize based on self-reported barriers to pain management. Also facilitates patient-provider communication through direct call button and provider access to patient-entered information.
Alnosayan et al (2017) [22]	MyHeart	Symptom management	Patients with heart failure	Symptom tracking and reminders for missing data available through app and sensor technologies. Educational and motivational messages sent daily.
Athilingam et al (2016) [23]	HeartMapp	Symptom management	Patients with heart failure	Offers symptom and vitals tracking through app and sensor technologies, exercises, and heart failure educational resources
Cox et al (2018) [24]	PCplanner	Decision support	Convoy	Family members of patients admitted to the intensive care unit can access palliative care educational resources and complete a needs assessment. Electronic health record triggers allow provider to approve palliative care consultation for patients based on health status and family request through app.
Foster (2018) [25]	HF App	Symptom management	Patients aged >50 years with heart failure	Offers daily symptom and vitals tracking and heart failure educational resources.
Hardinge et al (2015) [26]	Not applicable	Symptom management	Patients aged >50 years with *chronic obstructive pulmonary disease*	Offers symptom and well-being diary, including optional recording of medication use, collected through app and sensor technologies. Personalized plans for self-management and educational material also included.
Moradian et al (2018) [27]	ASyMS	Symptom management	Patients with cancer	Offers functionalities for monitoring and managing chemotherapy-related toxicity with personalized risk prediction modeling and decision support. High-severity symptom reports alert clinicians.
Triantafyllidis et al (2015) [28]	SUPPORT-HF	Symptom management	Patients with heart failure	Offers symptom tracking and reports, educational material, and ability to communicate with clinicians through app.

### Elements of Palliative Care and Target User

The majority of apps (15 commercial and 8 research apps) primarily targeted symptom management, followed by decision support (5 commercial and 1 research app) and bereavement or grief (5 commercial apps). Commercially available apps were most commonly designed for patients (8 apps) or both patients and convoy (9 apps), whereas the majority of research apps (8 apps) were designed for patient use only. The research apps primarily targeted a specific condition (8 apps), cancer, heart failure, or Chronic Obstructive Pulmonary Disease, whereas commercial apps focused on a more general population providing functions for multiple symptoms, various psycho-social support, and health decision making resources.

### Convoy Composition and Size

Among commercially available apps, features allowing the patient to share app-generated materials, for example, advanced care directives or legacy projects, via email or SMS text message, were the most common (10 apps) in terms of patient-convoy relationship facilitated by the app. Several apps allowed convoy members to be independent users, either connected to the patient and other convoy members (4 commercial apps and 1 research app) or not (3 commercial apps). An additional 5 commercial apps and the majority of research apps (8 apps) did not have a convoy component and targeted the patient only.

### Consideration of Personal and Contextual Characteristics

Several apps (5 commercial and 1 research) gave consideration to personal characteristics including age, gender, race, and ethnicity of the patient and/or convoy members. Few apps (2 commercial) considered contextual factors such as marriage, social isolation, or socioeconomic status of patient or convoy members. Furthermore, only 1 app included both personal and contextual characteristics.

## Discussion

### Principal Findings

mHealth may offer a simple, cost-effective method for keeping individuals connected and involved in care across the course of serious illness. This scoping review described the support for access and use by social convoy members in mHealth apps for palliative care. Overall, this review identified strengths, weaknesses, and areas for future work in mHealth for palliative care.

Results identified 14/22 (64%) commercial apps and 1/9 (11%) research app that included convoy members; however, only 5 in total, all of which were commercial apps, targeted convoy members as primary users. The vast difference in convoy-inclusion results yielded from research-based versus commercial apps suggests that the mHealth market considers caregivers consumers of their product, whereas researchers are focusing on patient-directed care. Although caregivers may be increasingly seen as potential users among developers, a recent review indicates that there are few commercial apps available specifically for caregiving [29] and limited usability evidence for caregiver apps [30]. Even though the inclusion of convoys in multiple apps was promising, convoy members were often passive recipients of information, and few apps targeted convoy-specific issues.

Many of the apps included in this review allowed patients to share information with convoy members regarding symptom management, decision making, and preferences. A strength of this feature is that sharing information may help convoy members understand symptom presentation, burden, and management. This is important in palliative care as families often struggle to identify the presence or severity of key symptoms (eg, pain or psychological distress) [31]. In addition, quick and convenient access to documentation of proxies and patient preferences may help establish consistency in decision making, promote confidence in time-limited decisions, and decrease decisional conflict. However, more research is needed to test the effect of sharing health information with social convoys on positive palliative care outcomes, including quality of life, symptom management, and goal concordant care.

Commercial apps offered tools and resources for a general audience, whereas research apps targeted specific illnesses. Often people with palliative care needs have more than one condition, and disease specific apps may be too limited. However, some mobile interventions may require specificity to address disease specific symptoms and improve health decision making. For example, illness trajectories differ by condition. Therefore, preparing advance directives or goals of care in the setting of heart failure can be different than treatment options for cancer. This specificity may not be available in more generalized palliative care apps.

A limitation of the symptom management and decision-making apps included in this review is that, typically, convoy members were passive recipients of information (ie, a 1-way flow of information from patient to convoy). Although patients’ ability to manage the dissemination of information may promote choice and control, there are also limitations to this structure. For example, cognitive impairment, pain, and/or fatigue may hinder motivation or ability to share information with convoy members, which may result in misinformation or conflict. In addition, ideally, palliative care follows a team-based approach with information continually flowing to and from patients, providers, and convoys rather than a single party disseminating information to others. Additional work needs to be done to investigate mHealth methods that support a patient-centered model of care and empower active involvement from convoy members.

The individual, social, or care-related outcomes of a hub (patient) and spoke (convoy members) model of flow of information, consistent with many of the apps included in this review, are unknown. As mentioned, this model may increase choice and control but might also relate with perceived sense of burden for patients. For example, sharing daily pain levels may increase the patient’s perceived burden on others and/or convoy members’ sense of helplessness. Future research is needed to explore relationships between mHealth use, convoy contact, perceived burden, and other salient outcomes (eg, relationship quality, satisfaction with care, or quality of life).

Coping with bereavement and grief was the most common palliative care component of apps targeting convoy members as primary users. Although grief occurs across the course of serious illness [32], the majority of apps were tailored to coping with bereavement after the death of the patient. These apps included common coping strategies such as connecting with social support, resource sharing and education, affirmations, and tracking behaviors or rituals. Accepted models of bereavement and grief suggest that individuals cope with stressors related to loss (ie, negative emotions) and restoration (ie, negotiating new roles) [33]. However, most of the apps included in this review focused on coping with loss-related stressors only, omitting any focus on restoration. Therefore, current mHealth apps for palliative care may not offer comprehensive coping tools for bereavement and grief and may be most useful early in the bereavement process (ie, soon after the death of a loved one). Future work needs to investigate how convoy members use mHealth apps for bereavement and grief and the impact of such app use on the coping process.

Few mHealth apps included in this review targeted convoy-specific issues other than bereavement and grief. Additional convoy-specific issues in palliative care not addressed by apps in this review include: care roles (eg, defining and providing education on roles among convoy members), skills and coping (eg, time management, stress reduction, or anticipatory grief), and communication (eg, planning family meetings, conflict resolution, or assertiveness training). The aforementioned topics, however, are not a comprehensive list of convoy-specific issues in palliative care, and engaging stakeholders may help developers and researchers design improved apps to meet the specific needs of this population.

A minority of apps included in this review considered convoy characteristics in development. Personal factors such as age, socioeconomic status, marriage, and social support likely influence preferences, needs, and usability for mHealth apps and, thus, should be considered in future works. Distance and relationship quality are also important convoy characteristics that were not considered in any app included in this review. mHealth may be particularly useful for facilitating involvement and meeting the needs of long-distance convoy members; however, this population is rarely given unique consideration in the development of interventions. Attention to relationship quality and conflict among patients, convoy members, and providers may be another important area to consider in future mHealth interventions. For example, apps may choose to offer settings and features to control the flow of information based on the quality of relationship, or offer strategies to address interpersonal conflict. Taken together, developers and researchers should appreciate the heterogeneity of convoy members and populations with unique needs, as this may impact needs, usage, and outcomes.

This review identified multiple areas for future app development and research in mHealth for palliative care. First, there is a need to consider team-based apps that promote active roles and flow of information among patients, convoy members, and providers. Second, few apps considered convoy-specific needs or characteristics, which may limit the reach and benefit of mHealth in palliative care. Next, many apps targeted individuals already connected to palliative services but did not address the potential of mHealth to help promote access to palliative care. Additional apps are needed to provide education and resources for connecting individuals with serious illness and their convoys with palliative care services. Finally, the apps included in this review isolated specific components of palliative care, possibly increasing user burden and/or decreasing usage by requiring various apps to meet palliative care needs. Additional work is needed to develop holistic mHealth apps consistent with the comprehensive palliative model of care (ie, biopsychosocial-spiritual).

Only 1 research app identified in this review included convoy members in any way, and that app did not target convoy members as primary users. The omission of convoys in research limits empirical knowledge on the reach and impact of mHealth in palliative care and raises questions regarding possible research challenges with this population. Previous scholars suggest there are unique challenges related to research with patients with serious illness and their care networks [34], but it is possible that app-based data collection may actually alleviate some of these issues (eg, fewer recruitment challenges). Therefore, it is unknown whether the scientific mission of mHealth apps in palliative care has narrowly focused on patients or if there are methodological limitations that create barriers for including this population in research. Additional work is needed to address best practices for mHealth research in palliative care, particularly related to the inclusion of social convoys.

### Strengths and Limitations

To the knowledge of the authors, this scoping review is the first to address the inclusion and function of social convoys in commercial and research mHealth apps for palliative care. As a result, our findings and interpretations may be instrumental for guiding future work in mHealth for palliative care, an emerging area of industry and science. However, there are some limitations to highlight. First, this review only included free commercially available apps from 3 major app stores, which may have biased findings. Furthermore, as apps identified in the research were unavailable commercially, review of research-based apps relied solely on the description of the app in the publication. Second, the scope of review did not consider mHealth solutions other than mobile apps. There are many palliative care digital health initiatives, including telemedicine, websites, and text message programs, currently underway to expand access to palliative care. However, mobile apps have the ability to offer palliative care tools and functions with and without access to the internet. Third, this review considered both commercial apps and apps developed in research settings and reported on features and functions available in the resulting products but did not assess the presence or strength of the evidence base with regard to the impact of any of the apps on their intended outcomes. In the absence of an evidence base, it is difficult to compare apps, and are therefore limited to describing the functions of palliative care apps rather than assessing quality.

### Conclusions

This scoping review highlighted important information on the inclusion and functionality of social convoy members in mHealth apps for palliative care. Results suggest there is an emerging presence of apps for patients and convoy members receiving palliative care; however, there are many needs for developers and researchers to address in the future. Specifically, additional work is needed for apps that embrace a team approach to information sharing, target convoy-specific issues, promote access to palliative care, and are comprehensive of palliative needs. Furthermore, the inclusion of convoys in mHealth research is severely lacking and requires attention in the literature. Limitations and recommendations presented in this review may help guide future development of mHealth apps and scientific studies designed to support the needs of patients and convoy members in palliative care.

## Abbreviations

mHealth: mobile health
PRISMA-ScR: Preferred Reporting Items for Systematic Reviews and Meta-Analyses extension for Scoping Review
